# Integration of enteric fever surveillance into the WHO-coordinated Invasive Bacterial-Vaccine Preventable Diseases (IB-VPD) platform: A low cost approach to track an increasingly important disease

**DOI:** 10.1371/journal.pntd.0005999

**Published:** 2017-10-26

**Authors:** Senjuti Saha, Maksuda Islam, Mohammad J. Uddin, Shampa Saha, Rajib C. Das, Abdullah H. Baqui, Mathuram Santosham, Robert E. Black, Stephen P. Luby, Samir K. Saha

**Affiliations:** 1 Child Health Research Foundation, Department of Microbiology, Dhaka Shishu (Children) Hospital, Dhaka, Bangladesh; 2 Department of International Health, Johns Hopkins Bloomberg School of Public Health, Baltimore, MD, United States of America; 3 Division of Infectious Diseases and Geographic Medicine, Stanford University School of Medicine, Stanford, CA, United States of America; 4 Bangladesh Institute of Child Health, Dhaka Shishu (Children) Hospital, Dhaka, Bangladesh; Duke University Medical Center, UNITED STATES

## Abstract

**Background:**

Lack of surveillance systems and accurate data impede evidence-based decisions on treatment and prevention of enteric fever, caused by *Salmonella* Typhi/Paratyphi. The WHO coordinates a global Invasive Bacterial–Vaccine Preventable Diseases (IB-VPD) surveillance network but does not monitor enteric fever. We evaluated the feasibility and sustainability of integrating enteric fever surveillance into the ongoing IB-VPD platform.

**Methodologies:**

The IB-VPD surveillance system uses WHO definitions to enroll 2–59 month children hospitalized with possible pneumonia, sepsis or meningitis. We expanded this surveillance system to additionally capture suspect enteric fever cases during 2012–2016, in two WHO sentinel hospitals of Bangladesh, by adding inclusion criteria of fever ≥102°F for ≥3 days, irrespective of other manifestations. Culture-positive enteric fever cases from in-patient departments (IPD) detected in the hospital laboratories but missed by the expanded surveillance, were also enrolled to assess completion. Costs for this integration were calculated for the additional personnel and resources required.

**Principal findings:**

In the IB-VPD surveillance, 5,185 cases were enrolled; 3% (N = 171/5185) were positive for microbiological growth, of which 55% (94/171) were culture-confirmed cases of enteric fever (85 Typhi and 9 Paratyphi A). The added inclusion criteria for enteric fever enrolled an additional 1,699 cases; 22% (358/1699) were positive, of which 85% (349/358) were enteric fever cases (305 Typhi and 44 Paratyphi A). Laboratory surveillance of in-patients of all ages enrolled 311 additional enteric fever cases (263 Typhi and 48 Paratyphi A); 9% (28/311) were 2–59 m and 91% (283/311) >59 m. Altogether, 754 (94+349+311) culture-confirmed enteric fever cases were found, of which 471 were 2–59 m. Of these 471 cases, 94% (443/471) were identified through the hospital surveillances and 6% (28/471) through laboratory results. Twenty-three percent (170/754) of all cases were children <2 years. Additional cost for the integration was USD 44,974/year, a 27% increase to the IB-VPD annual expenditure.

**Conclusion:**

In a setting where enteric disease is a substantial public health problem, we could integrate enteric fever surveillance into the standard IB-VPD surveillance platform at a modest cost.

## Author summary

Typhoid/paratyphoid fever imposes a major global burden, specifically in low-and-middle-income countries (LMICs). However, it is challenging to implement evidence-based decisions for treatment and prevention because of lack of data from comprehensive surveillance systems, which are often expensive and difficult to sustain. The WHO has established a global surveillance program called “Invasive Bacterial–Vaccine Preventable Diseases (IB-VPD) Surveillance” to capture sepsis, meningitis and pneumonia in under-five children in many LMICs. Data generated by this program have facilitated introduction of live-saving vaccines and development of treatment strategies. However, the program does not include typhoid/paratyphoid surveillance. We tested the feasibility and sustainability of integrating typhoid/paratyphoid surveillance into this program in two leading children’s hospitals in Bangladesh. By monitoring all patients with signs of typhoid/paratyphoid, we captured 471 laboratory-confirmed episodes in under-five children between Jan 2012 and Dec 2016. Blood culture results from all in-patients revealed that the proposed expanded surveillance captures 94% of hospitalized typhoid/paratyphoid cases. Thirty-six percent (170/471) of 2–59 m cases were in children <2 years. Overall, age distribution and antibiotic resistance patterns were consistent with data generated from larger, expensive and typhoid-specific surveillance programs in the region, adding credence to the proposed integration. Adding typhoid/paratyphoid surveillance to an established invasive disease surveillance platform took advantage of existing infrastructure and resources and as such was easy and cost-effective to implement. We recommend that WHO considers similar integration in other countries; data generated from such surveillances will help countries make evidence-based decisions on introduction of upcoming vaccines and prepare for impact studies.

## Introduction

Enteric fever (typhoid/paratyphoid) is a major cause of mortality and morbidity in many low and middle-income countries. In 2015, it was estimated to cause about 178,000 deaths and 17 million illnesses; 85% of all cases occur in three countries—Bangladesh, India and Pakistan [1–3]. The primary causative agents of enteric fever are *Salmonella enterica* serovars Typhi (typhoid) and Paratyphi A, B, and C (paratyphoid) [4]. Enteric fever was one of the largest killers during the pre-antibiotic era, but case fatality rates have decreased from 30% to less than 1% with the use of effective antibiotics [4]. In recent years, however, there has been an increasing number of reports on the rise of antimicrobial resistance of *Salmonella* Typhi/Parathyphi [5]. If this trend is not interrupted, untreatable infections with case fatality rates much higher than those experienced in the last few decades are likely to occur. This evolving crisis calls for urgent guidelines for institution of effective treatment and prevention policies; however, a scarcity of accurate data on burden and epidemiological and antimicrobial resistance trends impedes evidence-based policy decisions. For example, despite the availability of the typhoid Vi polysaccharaide vaccines (ViPS) that provide protection in >2 years children in Bangladesh, where the main etiology of blood stream infections is *Salmonella* Typhi/Paratyphi A [6–8], they are not used programmatically. Due to poor reception by the public and clinicians, two pharmaceutical manufacturers ultimately removed their products from market; consumption in 2013 was only about 2,500 doses (personal communication). Patients and physicians largely rely on empirical antibiotic therapy. This lack of data primarily stems from a lack of robust surveillance for enteric fever in endemic areas, which are mainly in resource-poor countries that cannot afford to establish and/or sustain new surveillance programs for this disease [9]. Credible disease estimates can help policy-makers and government officials to prioritize interventions.

Unlike for enteric fever, most countries already have, or are in the process of introducing, pneumococcal conjugate vaccine (PCV) and *Haemophilus influenzae* type b (Hib) vaccine to combat invasive diseases caused by *Streptococcus pneumoniae* and Hib, respectively. The decisions to implement these vaccines have been facilitated by surveillance data generated by the Gavi’s PneumoADIP and Hib Initiative and the World Health Organization (WHO)-coordinated Global Invasive Bacterial-Vaccine Preventable Diseases (IB-VPD) Surveillance Network. The IB-VPD surveillance assesses the burden of pneumonia, meningitis and sepsis in children ≤59 months. This surveillance system captures data on *S*. *pneumoniae*, Hib and *Neisseria meningitidis* (from blood and cerebrospinal fluid, CSF) and monitors long-term trends, including the impact of vaccines [10]. As of 2016, the system is in place in 56 countries [11]. Notably, enteric fever is not part of this surveillance platform.

Since 2009, Bangladesh has been operating four high-performing WHO IB-VPD sentinel surveillance sites in Dhaka (two sites), Chittagong and Mirzapur. Epidemiological data generated from these sites facilitated introductions of Hib and pneumococcal vaccines in Bangladesh [11]. In this study, we aimed to evaluate the logistics, cost and sustainability of leveraging the ongoing WHO-coordinated IB-VPD platform for enteric fever surveillance by broadening the inclusion criteria of the original surveillance.

## Methods

### Study site and population

The study was conducted in two urban hospitals, Dhaka Shishu (Children) Hospital (DSH) and Shishu Shasthya (Child Health) Foundation Hospital (SSFH), which are sentinel sites of the WHO IB-VPD platform in Bangladesh. These are the two largest pediatric hospitals in the country. DSH provides primary to tertiary care to patients aged 0–18 years; it acts both as a primary point of care and the major referral hospital of the country. SSFH acts as the primary point of care for children aged 0–14 years. Together, they have 840 beds, 246 (29%) of which are reserved for families who are unable to pay. The hospitals are located close to each other and primarily cater to the same catchment area of Mirpur, Dhaka.

### Patient enrollment

In Bangladesh, using the WHO-coordinated IB-VPD surveillance system, we monitor pneumonia, meningitis and sepsis in children under 5 years old admitted in the in-patient departments (IPD) using protocols described elsewhere [12]. In brief, IPD patients are assessed by research physicians and are considered eligible for WHO IB-VPD surveillance study if they meet clinical definitions for meningitis, pneumonia or sepsis (WHO’s IB-VPD inclusion criteria, Table 1) [12]. Blood specimens are collected from eligible cases at the discretion of the attending physicians. Only cases from which a specimen is collected are enrolled. In January 2012, we expanded the existing surveillance platform to include surveillance of enteric fever; based on WHO case definition, we set inclusion criteria for enrollment as fever of ≥102°F for ≥3 days with no clinical manifestations of pneumonia, meningitis or sepsis (Table 1) [13]. Here we report our findings from this expanded surveillance between January 2012 and December 2016.

**Table 1:** Inclusion criteria for the WHO-coordinated IB-VPD surveillance system and the proposed additional enteric fever surveillance.

Inclusion criteria of IB-VPD surveillance system			Added inclusion criteria for enteric fever surveillance
Meningitis	Pneumonia	Sepsis	Enteric Fever
sudden onset of fever of >100.4°F (axillary) and one of the following signs: neck stiffness, altered consciousness with no other alternative diagnosis, or other meningeal sign	coughing or difficulty breathing and tachypnea when calm at a rate of ≥60 breaths/min in an infant aged <2 months, ≥50 breaths/min in an infant aged 2 to <12 months, or ≥40 breaths/min	presence of at least two of the following danger signs and without meningitis or pneumonia: inability to drink or breastfeed, vomiting everything, convulsions (except in malaria endemic areas), prostration/lethargy (abnormally sleepy or difficult to wake), severe malnutrition and hypothermia (≤96.8°F)	presence of fever of ≥102°F (axillary) for at least 3 days in a child with or without any other clinical manifestation

To test the success rate of capturing enteric fever cases using the expanded IB-VPD platform, we additionally enrolled all cases (of any age and any clinical presentation) from the IPD whose blood culture yielded growth of *Salmonella* Typhi/Paratyphi A in the laboratories but were not enrolled in the active hospital surveillance systems. Microbiology laboratories of the two hospitals are also the diagnostic laboratories that provide service to all patients. They receive and culture all blood specimens referred by physicians in the hospitals. Blood culture results of all cases were communicated to the study physicians by the laboratory staff as soon as they were available. Study physicians at DSH and SSFH reviewed these cases of *Salmonella* Typhi/Paratyphi A that were not captured through the IB-VPD or enteric fever surveillance systems and enrolled them in the study. Clinical information of these culture-confirmed enteric fever cases were collected through the study physicians’ assessment within 24 hours of laboratory confirmation from hospital charts [12].

### Etiology detection and antibiogram

Blood cultures were performed using standard methods as described earlier [14]. In brief, 2–3 ml blood was aseptically obtained and inoculated into trypticase soy broth supplemented with sodium polyethanol sulphonate (0.25%) and isovitalex (1%). All blood collections were performed by trained hospital phlebotomists and stringent measures were taken to avoid contamination by skin flora during phlebotomy. Blood collection was only performed once per patient. Incubated blood culture bottles were sub-cultured on the 2^nd^, 3^rd^ and 5^th^ days of incubation. Identification of *Salmonella* Typhi/Paratyphi A isolates was confirmed after standard biochemical tests and agglutination with *Salmonella* species and serovar specific antisera (Ramel, Thermo Fisher Scientific, USA). Antibiotic susceptibility tests were conducted and interpreted according to most the recent Clinical and Laboratory Standards Institute (CLSI) guidelines for each antibiotic. Disc diffusion methods were used for ampicillin, cotrimoxazole, chloramphenicol, ciprofloxacin, azithromycin and ceftriaxone. In addition, microbroth dilution method was performed for ciprofloxacin.

### Data analysis

All data were entered into EpiData (The EpiData Association, Odense, Denmark) and analyzed using Stata 13 (STATACorp, College Station, TX). Data were analyzed to examine the distribution of culture-positive etiological agents of meningitis, sepsis, pneumonia and enteric fever cases, and their clinical and epidemiological features. Age distribution and antimicrobial susceptibility of enteric fever cases were also examined. No statistical tests were performed in this study.

### Cost calculations

The cost of integrating enteric fever surveillance into the ongoing IB-VPD surveillance was calculated based on the wet-laboratory resources required to process 1,699 additional blood specimens and the additional personnel appointed. While calculating wet laboratory expenses, extra items required for culture and identification of *Salmonella* Typhi and *Salmonella* Paratyphi A using biochemical and serological tests with specific antisera were considered. Two research physicians (50% time each, one at each facility) and two research assistants (50% time each, one at each facility) were needed to aid in assessment of the additional cases that became eligible and were enrolled due to the additional enteric fever surveillance inclusion criteria.

### Ethical clearance

The protocols were approved by the ethics review committees of the Bangladesh Institute of Child Health, Dhaka Shishu (Children) Hospital, Bangladesh. Blood samples were collected as part of routine clinical care and written consent was obtained from parents or caregivers of all participants for other aspects of the study, including collection of data and use of specimens for additional laboratory analysis.

## Results

### Patient enrollment through the original IB-VPD surveillance and the added enteric fever criteria

**Fig 1:**
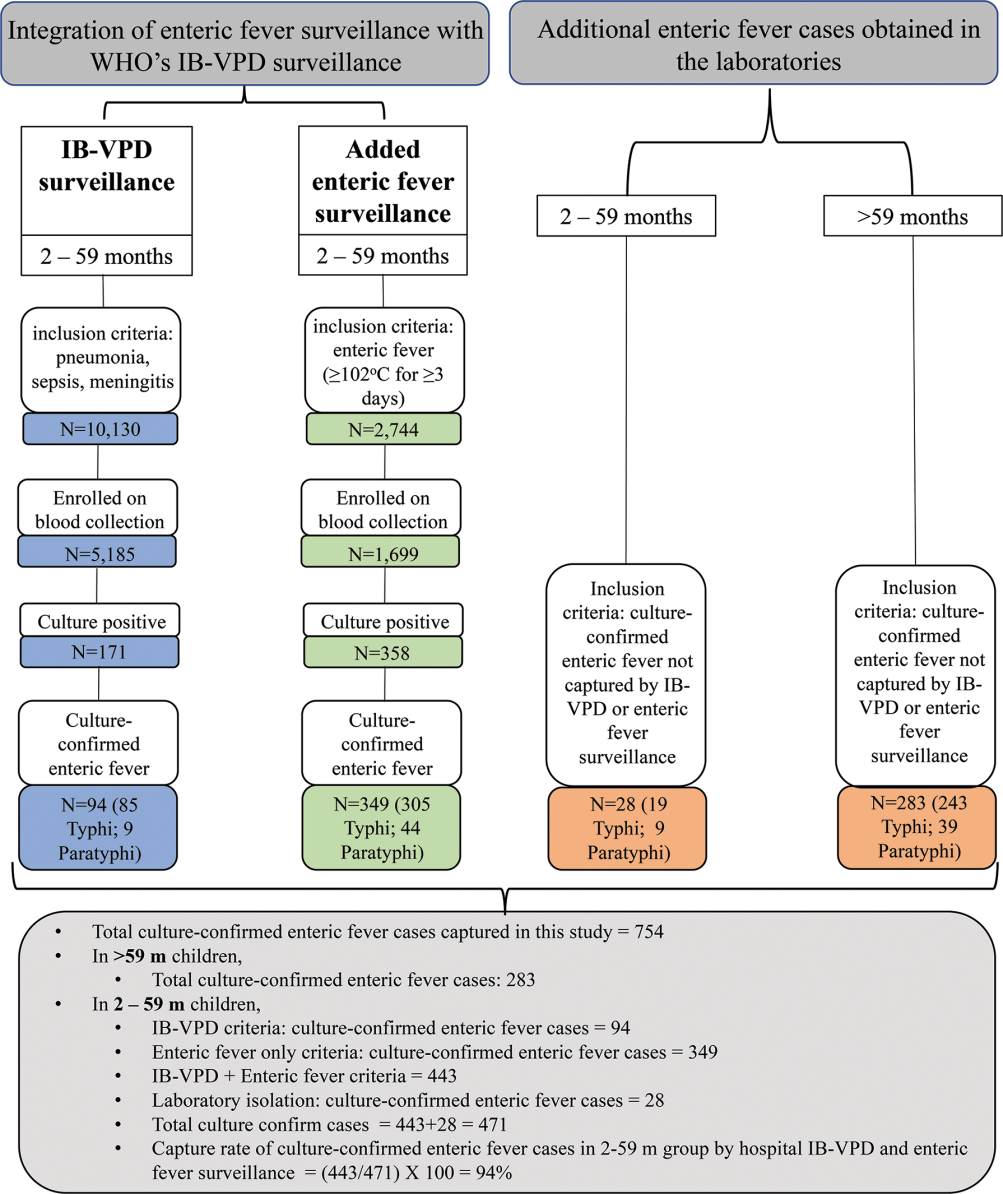
Overview of integration of enteric fever surveillance into the WHO-coordinated IB-VPD surveillance system.

**Fig 2:**
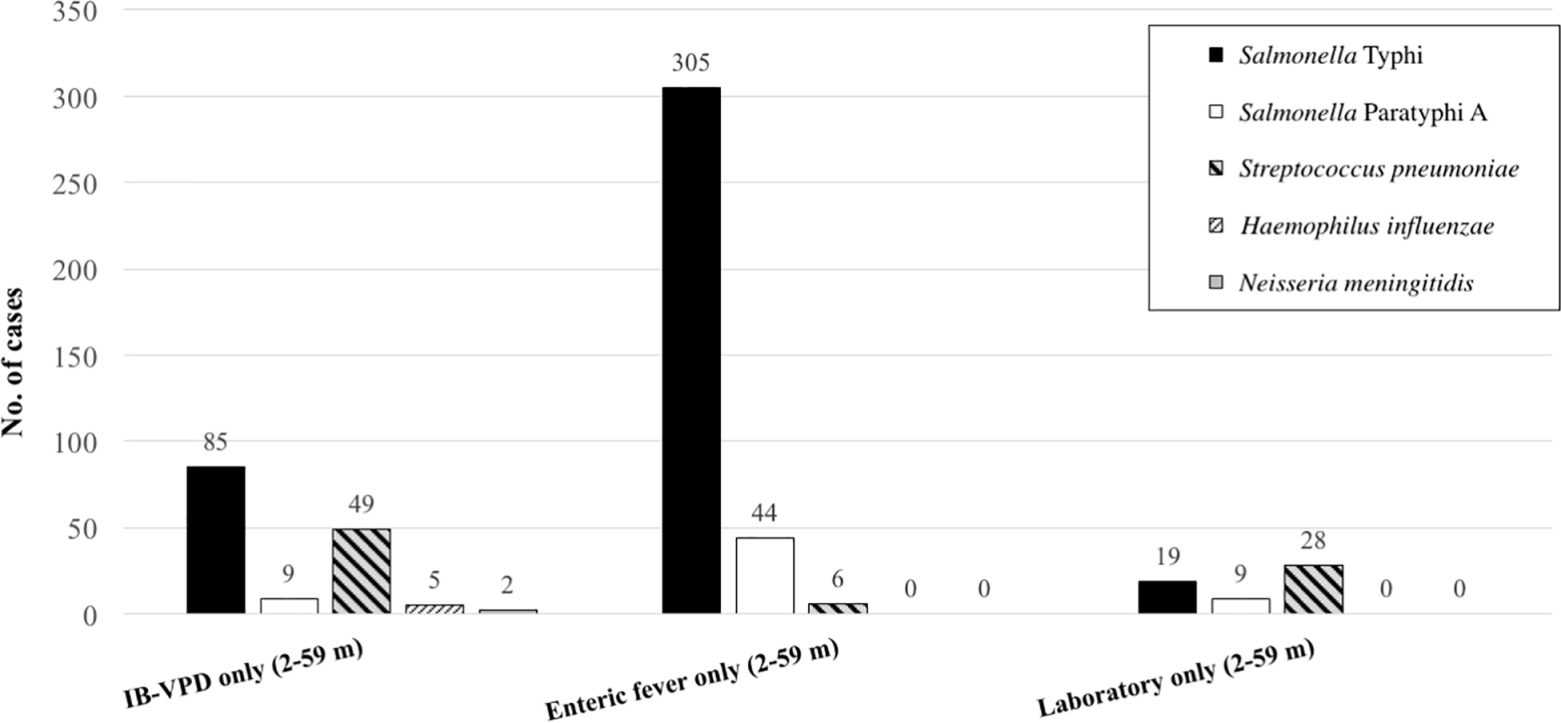
Predominant bacterial etiologies isolated in the hospital and laboratory surveillance systems.

Based on the original inclusion criteria defined by the WHO-coordinated IB-VPD surveillance, 10,130 cases were identified with possible invasive bacterial diseases (sepsis, pneumonia, meningitis) (Fig 1). Amongst them, 5,185 (52%) cases were enrolled on collection of blood specimen for culture; 171 (3.3%, 171/5,185) cases were culture-positive (Fig 1). Of the organisms detected, 55% (94/171) were either *Salmonella* Typhi (50%; 85/171) or *Salmonella* Paratyphi A (5.2%; 9/171). The other predominant organisms were *S*. *pneumoniae* (29%; 49/171) and *N*. *meningitidis* (1.2%; 2/171) (Fig 2). A full list of organisms detected in this original platform are listed in S1 Table.

Using the added inclusion criteria of ≥102°F for ≥3 days for enteric fever, a further 2,744 cases with suspect enteric fever were identified, who did not meet the clinical criteria of WHO-defined meningitis, sepsis or pneumonia (IB-VPD surveillance). Blood specimens were collected from 1,699 (62%, 1699/2744) of them and a total of 358 (21%, 358/1699) specimens were culture-positive. Amongst the culture-positive cases, *Salmonella* Typhi was the predominant etiology (85%, 305/358), followed by *Salmonella* Paratyphi A (12%, 44/358) (Fig 1). Thus, the additional case definition increased the number of all blood cultures performed by 25% {1699/(1699+5185)}and this resulted in a five-fold increase in the detection of enteric fever cases (from 94 to 443 in five years). Of the remaining nine culture-positive specimens captured with the additional inclusion criteria, six were cases of *S*. *pneumoniae* infections, two of non-typhoidal *Salmonella* infections and one of *Escherichia coli* infection (Fig 2, S1 Table).

### Evaluation of enteric fever surveillance on WHO IB-VPD platform

In all age groups seeking care at the in-patient departments of the hospitals, a total of 311 culture-confirmed enteric fever cases (263 *Salmonella* Typhi and 48 *Salmonella* Paratyphi A) cases were identified in the laboratories that were not enrolled through the WHO IB-VPD surveillance or the added enteric fever surveillance. Amongst them, 9% (28/311) were from children 2–59 months old, the WHO IB-VPD age group. The combination of the IB-VPD and the added fever ≥ 102°C for ≥ 3 days in the case definition captured 443 cases in this age group. Thus, the proposed expanded surveillance, using the IB-VPD platform, captured 94% (443/471) of the blood culture-confirmed enteric fever cases among 2–59 months old in-patients. The remaining 91% (283/311) culture-confirmed enteric fever cases obtained in the laboratories were from children > 59 m. In total, 754 culture-confirmed typhoid and paratyphoid cases were enrolled from the in-patient departments in this study. Of them, 283 (38%) cases were children aged > 59 m and hence only captured after obtaining laboratory results.

**Fig 3:**
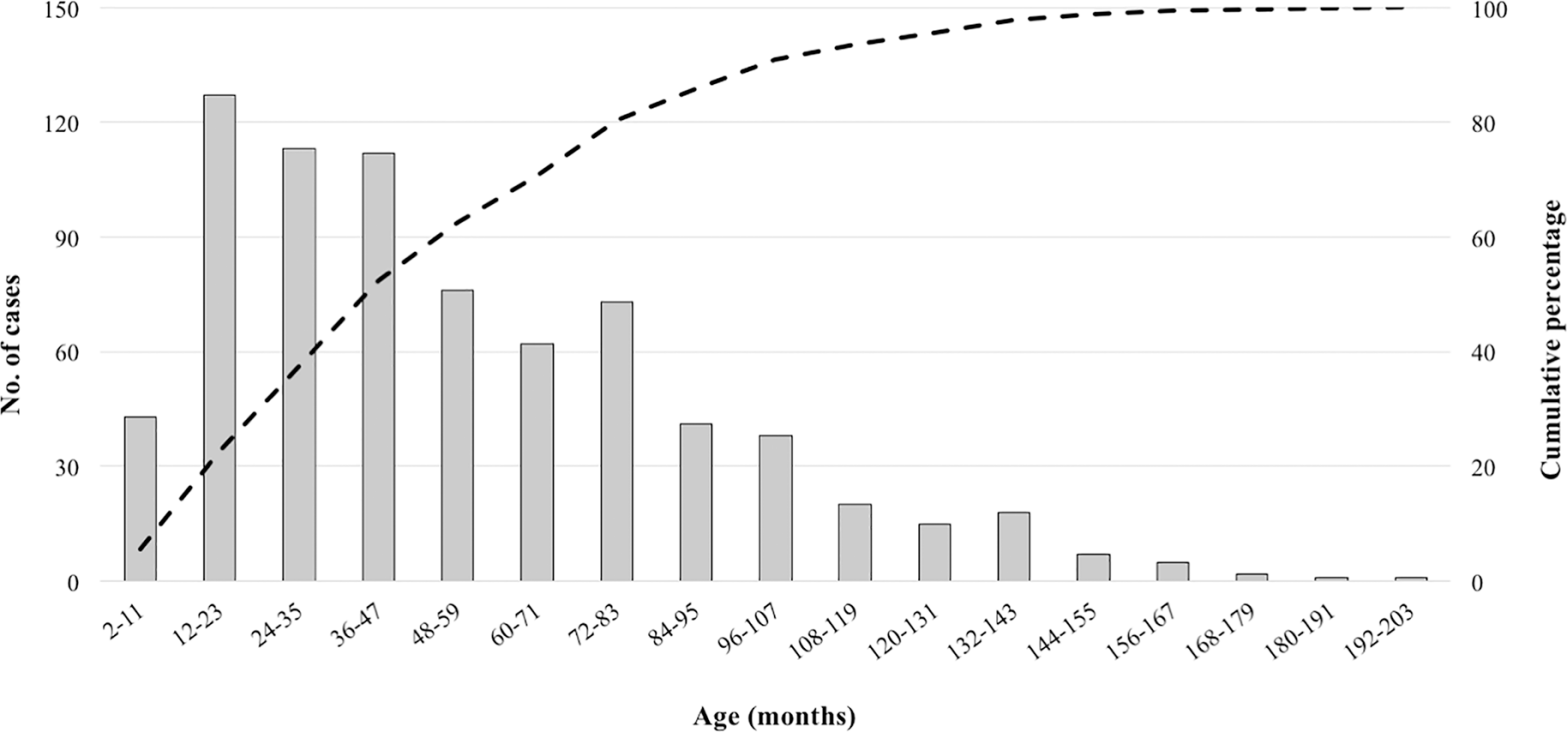
Age distribution of culture-positive enteric fever cases in children hospitalized in DSH and SSFH, captured using the proposed expanded IB-VPD surveillance system from Jan 2012 to Dec 2016.

Out of 754 total culture-positive typhoid/paratyphoid cases identified, 471 enteric fever infections were in the target IB-VPD population of 2–59 m old children. In this age group, 36% (170/471) of the infections occurred in the first 24 months of life. Children in their second year of life seemed most vulnerable with 27% (125/471) cases occurring in children aged 12–23 months (Fig 3).

### Antimicrobial resistance profiles

Antibiotic susceptibility was tested for all *Salmonella* Typhi (N = 653) and *Salmonella* Paratyphi A isolates (N = 101). In total, 166 (25%) *Salmonella* Typhi isolates were multi-drug resistant (MDR, resistant to chloramphenicol, ampicillin and trimethoprim). No MDR *Salmonella* Paratyphi A strain was identified. While 578 (89%) of *Salmonella* Typhi and 70 (69%) of *Salmonella* Paratyphi A isolates were susceptible to azithromycin, all strains were susceptible to ceftriaxone. By contrast, 646 (99%) *Salmonella* Typhi and 99 (99%) *Salmonella* Paratyphi A isolates were non-susceptible to ciprofloxacin.

### Clinical manifestation of laboratory-confirmed enteric fever cases

Clinical manifestations of all 754 laboratory-confirmed typhoid and paratyphoid cases were assessed (Table 2). Of the 94 cases captured in the original WHO IB-VPD surveillance, 78% (73/94) had fever of ≥102°F for ≥3 days. Median duration of fever was five days. Other manifestations included vomiting (30%, 28/94), convulsions (37%, 35/94) and inability to feed (17%, 16/94). In the 349 cases captured with the added inclusion criteria for enteric fever, where only children with ≥ 102°C fever for ≥ 3 days were enrolled, the median duration of fever was six days and vomiting (37%, 130/349) was often observed. Overall, clinical manifestation of enteric fever cases identified by the IB-VPD surveillance appeared more severe than those identified based on expanded fever ≥ 102°C fever for ≥ 3 days criteria, most notably with convulsions (37% vs 4%) and inability to feed (17% vs 0%) occurring at a higher rate.

**Table 2:** Distribution of clinical signs amongst confirmed enteric fever cases (N = 754).

Clinical Manifestations	Confirmed enteric fever cases captured in the IB-VPD platform (N = 94)	Enteric fever cases by added definition (≥102°F for ≥3 days) (N = 349)	Enteric fever cases in 2–59 m old cases enrolled through laboratory confirmation (N = 28)	Enteric fever cases in >59 m old cases enrolled through laboratory confirmation (N = 283)
Median fever duration (days)	5	6	6	6
Temperature (≥100.4°F)	94 (100%)	349 (100%)	9 (32.1%)	274 (96.8%)
Temperature (≥102°F)	88 (93.6%)	349 (100%)	1 (3.6%)	249 (88.0%)
Fast breathing	2 (2.1%)	0 (0.0%)	0 (0.0%)	0 (0.0%)
Chest indrawing	0 (0.0%)	0 (0.0%)	0 (0.0%)	0 (0.0%)
Convulsion	35 (37.2%)	4 (1.1%)	2 (7.1%)	3 (1.1%)
Inability to feed	16 (17.02%)	0 (0.0%)	0 (0.0%)	9 (3.2%)
Vomiting	28 (29.8%)	130 (37.2%)	9 (32.1%)	117 (41.3%)

Amongst the 28 2–59 m children enrolled after laboratory confirmation, who were neither captured by IB-VPD or enteric fever surveillance, physicians observed a fever of ≥102°F in only one case and the duration of fever was <3 days. Vomiting was the most common manifestation (32%, 9/28). In the >59 months-old group (N = 283), fever of ≥102°F was observed in 249 (88%) cases and the median duration of fever was six days. The most common accompanied clinical sign was also vomiting (41%, 117/283).

### Modest additional costs and resources were required to leverage the IB-VPD surveillance for enteric fever

The additional cost for running the enteric fever surveillance leveraging the WHO’s IB-VPD platform was USD 19,374 per year for research physicians and assistants, in addition to the base expenditure of USD 167,765 of the platform. The wet laboratory work required additional reagents and resources of USD 25,600 per year. The total of USD 44,974 for added enteric fever surveillance is a 27% increase to the annual IB-VPD cost.

## Discussion

In South Asia, *Salmonella* Typhi/Paratyphi A comprise three-fourths of all isolates obtained from blood cultures of sick pediatric and general populations [6–8]. Enteric fever can be prevented by improving water, sanitation and hygiene and with effective vaccines. However, as it is a disease that disproportionately affects resource-poor communities, unless the vaccines are provided through the government health services, the new generation typhoid vaccine carries a risk of not protecting the most vulnerable children in the poorest countries. Moreover, it remains difficult for policy makers to make evidence-based decisions as historically only a few, small, sporadic studies have addressed disease burden. Recently, with investments like the Coalition against Typhoid by the Bill and Melinda Gates Foundation, there has been renewed interest among the global community, including industries, in this disease. The Sabin Vaccine Institute, with support from the Bill and Melinda Gates Foundation, established a hospital-based enteric fever surveillance network in Asia called the Surveillance for Enteric Fever in Asia Project (SEAP), to enable systemic collection of data and fill knowledge gaps on impact of severe enteric fever. Another surveillance program, Typhoid Fever Surveillance in Africa Program (TSAP), was completed in Africa in 2014 and its follow-on study, Severe Typhoid in Africa (SETA) that measures the severity and burden of enteric fever, is underway [15]. However, such comprehensive population-based surveillance systems are expensive and bear the risk of unsustainability. For example, the large multi-country study, Pneumococcal Vaccine Accelerated Development and Introduction Plan (PneumoADIP), successfully demonstrated the burden of pneumococcal disease in developing countries and facilitated introduction of the appropriate vaccines [16]. Nevertheless, due to the high costs, this comprehensive surveillance system was not sustained. To sustainably monitor trends of enteric fever and the characteristics of its etiologies, sustainable and cost-effective surveillance systems are desirable. In Bangladesh, we have been able to establish a cost-effective integration of enteric fever surveillance within the ongoing WHO-coordinated IB-VPD surveillance system to generate data on another vaccine preventable disease.

During 2012–2016, in the IB-VPD surveillance, 55% etiologies of all blood culture positive cases of suspect pneumonia, sepsis and meningitis were *Salmonella* Typhi and Paratyphi A, despite the fact that IB-VPD does not aim to monitor enteric fever. Over the same time period, our attempt to identify additional enteric fever cases using added inclusion criteria of fever >102°F for ≥ 3 days increased capture of culture-confirmed enteric fever episodes in 2–59 m children from 19/year to 89/year. We report a total of 471 cases in this age group, where 94 were captured through the original IB-VPD surveillance and 358 through the added enteric fever surveillance. An additional 28 cases were found through analysis of laboratory results, which were missed by the inclusion criteria of the surveillance study. Overall, this integrated IB-VPD and enteric fever surveillances was able to capture 94% (443/471) of culture-confirmed enteric fever cases in 2–59 m children. This integration was sustainably managed for five years with no major hurdles and required an increase of 27% in cost; co-sharing of resources and personnel make the proposed surveillance a cost-effective approach.

The data generated from this multi-layered and multi-year study corroborate with results from previous typhoid specific studies from the region. Previous studies in Bangladesh showed that more than 50% of typhoid cases occur in children under the age of 5 years, similar to the findings of this study [7,17,18]. The rate of multidrug resistance in recent years has been reported to be around 20% for *Salmonella* Typhi strains in Bangladesh with more than 90% non-susceptibility to ciprofloxacin [6,19]. Similar rates were observed in our surveillance.

The surveillance system proposed here is not without limitations. Firstly, blood cultures were not performed for all suspect cases. This limitation is intrinsic to all IB-VPD sentinel site based surveillance systems since obtaining blood cultures is mainly dependent on the discretion of the treating physicians. To improve the blood-culture practice, our study team leveraged state-of-the-art laboratories and provided the test free of cost and the hospital authorities encouraged the treating physicians to advise blood culture. However, the large number of culture-positive cases (471 cases in 2–59 m patients and 283 cases in >59 m patients) that were detected yielded large amount of information and indicate that the proposed integrated surveillance system can be used to generate high quality epidemiological data and monitor antimicrobial resistance trends. Secondly, previous studies performed in Bangladesh and other endemic countries of the region show that majority of enteric fever cases seek care at the out-patient departments of hospitals, where they are commonly treated using empirical therapy; as the proposed enteric fever surveillance rides on the ongoing IB-VPD surveillance, only in-patient cases can be captured. Despite missing a large proportion of cases, such a surveillance program will capture the most severe cases with higher likelihood of hospitalization. Moreover, the antimicrobial resistance trends learnt from documented cases in this surveillance can guide empirical treatment policies in out-patient departments. Thirdly, because the surveillance reporting is not population-based, it does not have a denominator and thus does not allow for incidence calculation. This can be overcome if the data can be linked to a denominator using the low-cost hybrid approach proposed by Luby *et al* [9]. This approach combines the existing laboratory diagnosis data conducted in healthcare centers with those from community-based surveillance of utilization of healthcare facilities (study hospitals) to generate incidence estimates. We are currently initiating such a combined surveillance to calculate incidence and generate data relevant to policy decisions. Additional resources are also being invested to follow-up patients to characterize outcome and estimate disease severity and case fatality rates. Furthermore, previous work on impact of pneumococcal conjugate vaccine has shown that with such large number of cases in sentinel sites, it is also possible to monitor vaccine impact in hospital-based surveillance systems [20]. Fourthly, the stated costs for the proposed integration were estimated based upon sites that have considerable experience in bacterial disease surveillance. It is unclear how much this experience and these costs would transfer to other sites.

Overall, this study demonstrates that enteric fever surveillance can be sustainably and cost-effectively integrated into the original IB-VPD surveillance and the proposed integrated platform will fulfill the objectives of WHO for other invasive bacterial vaccine preventable diseases: (i) to collect data to describe epidemiology and estimate burden, (ii) to establish a surveillance platform in order to establish baseline rates of disease to measure impact after introduction of vaccines and (iii) to detect and characterize circulation bacterial types [10]. Establishment of a new and stable surveillance platform is time consuming and expensive and there is a high possibility of failure. With typhoid conjugate vaccines in sight we recommend that WHO considers the integration of enteric fever surveillance into the present IB-VPD platform. With such a system in place, areas with known high burden of enteric fever will benefit from better understanding of epidemiological characteristics of the disease and antimicrobial resistance trends for optimal empirical treatment. We also recommend this integration in areas where the burden of enteric fever is unknown, as this can act as a rapid and cost-effective way to monitor hospitalized children without major changes in infrastructure and or loss of resources if the disease is found to be absent. Data generated from such surveillance systems will help countries make evidence-based decisions on introduction of upcoming vaccines and prepare for evaluation of vaccine impact studies. By including enteric fever surveillance into the WHO-coordinated IB-VPD surveillance, robust data can be obtained about the true burden of one of the leading vaccine preventable diseases.

## Supporting information

S1 Table Etiologies found in blood specimens of cases enrolled in the IB-VPD and enteric fever surveillances systems. (DOCX)
