# Supplementary material for: Integration of enteric fever surveillance into the WHO-coordinated Invasive Bacterial-Vaccine Preventable Diseases (IB-VPD) platform: A low cost approach to track an increasingly important disease
Source: PLoS Negl Trop Dis. 2017 Oct 26;11(10):e0005999. doi: 10.1371/journal.pntd.0005999 (PMC5658195; doi:10.1371/journal.pntd.0005999)
Supplement: S1 Table — (DOCX) [file pntd.0005999.s001.docx]

| **Species** | **IB-VPD surveillance (N=171)** | **Enteric fever surveillance (N=358)** |
| --- | --- | --- |
| *Haemophilus influenzae* | 5 | 0 |
| *Streptococcus pneumoniae* | 49 | 6 |
| *Salmonella* Typhi | 85 | 305 |
| *Salmonella* Paratyphi A | 9 | 44 |
| *Salmonella sp.* | 1 | 2 |
| *Neisseria meningitidis* | 2 | 0 |
| *Acinetobacter sp.* | 3 | 0 |
| *Staphylococcus aureus* | 3 | 0 |
| *Klebsiella pneumoniae* | 4 | 0 |
| *Eshcherichia coli* | 4 | 1 |
| *Enterobacter sp.* | 0 | 0 |
| *Streptococcus sp.* | 0 | 0 |
| *Pseudomonas sp.* | 1 | 0 |
| *Serratia sp.* | 1 | 0 |
| *Streptococcus pyogenes* | 1 | 0 |
| *Group B Streptococcus* | 1 | 0 |
| *Flavobacterium sp.* | 1 | 0 |
| *Group D Streptococcus* | 1 | 0 |
| Total | 171 | 358 |
